# Creating a care pathway for patients with longstanding, complex eating disorders

**DOI:** 10.1186/s40337-022-00648-0

**Published:** 2022-08-29

**Authors:** Megan Reay, Joanna Holliday, John Stewart, Joanna Adams

**Affiliations:** 1 Oxford Health NHS Foundation Trust, Oxford, UK; 2 The Oxford Institute for Clinical Psychology Training and Research, Oxford, UK

**Keywords:** Longstanding eating disorder, Severe and enduring eating disorder (SEED), Service improvement, Treatment, Care pathway

## Abstract

**Background:**

Recovery rates for people with eating disorders are low; fewer than half recover and approximately 20% develop a longstanding eating disorder. Patients with longstanding eating disorders are often referred to as “SEED” (severe and enduing eating disorders) although this remains controversial and is not acknowledged in the British treatment guidance. This project aimed to generate recommendations for a longstanding eating disorder care pathway by identifying what proportion of patients have longstanding eating disorders and how to best identify and support them.

**Methods:**

Initially, a literature review was completed, followed by interviews with service-users who consider themselves to have longstanding eating disorders, and focus groups with staff members. The results were combined to create a definition of a longstanding eating disorder which was used to establish how many service-users could benefit from the pathway. The qualitative data was used to produce recommendations for a tailored pathway for those with longstanding eating disorders.

**Results:**

The results highlighted that, although “SEED” is often used, participants preferred to be referred to as “longstanding” or having no label. Qualitative analysis identified four themes in relation to supporting this population group which described how to structure the service and individualise care, as well as patients’ relationship to the service, and how to build a life after eating disorder services.

**Conclusions:**

Recommendations included promoting a hopeful message, focusing on quality of life and introducing peer support. Crucially, accessing the pathway should not result in being labelled “SEED”, nor should it prevent access to recovery focused interventions including weight restoration. The full list of recommendations are included as well as the implications of the project and limitations.

**Supplementary Information:**

The online version contains supplementary material available at 10.1186/s40337-022-00648-0.

## Plain English summary

It is known that as many as 20% of people with eating disorders do not recover, and go on to live with their eating disorder for a number of years. However, there is relatively little research or guidance for professionals about how to support this group of people. Therefore, this project aimed to design a pathway for patients with longstanding eating disorders by combining the research evidence, staff’s expert opinion and patient’s views. The results highlighted that the majority of participants in this sample expressed a dislike for the term ‘SEED’ (severe and enduring eating disorder) and preferred ‘longstanding eating disorder’ or having no label. The results were used to generate a set of recommendations about how services can best support this group of patients which covered how to structure the service, individualise care, manage patient’s relationship to the service, and build a life after eating disorder services. Key ideas included the importance of remaining hopeful about future recovery, introducing peer support, and supporting patients to improve their quality of life.

**Supplementary Information:**

The online version contains supplementary material available at 10.1186/s40337-022-00648-0.

## Background

### Existing literature

Eating disorder is an umbrella term for several diagnoses which are characterised by abnormal eating behaviours alongside a preoccupation with food, weight and body shape concerns [58]. In the UK, approximately 16% of adults screen positive for a possible eating disorder [31] and 1.25 million people are living with an eating disorder [8]. The impact of eating disorders is large, and the mortality rate high [4, 55], therefore, finding effective treatments is a priority. Despite the range of treatments recommended in the UK National Institute for Health and Care Excellence (NICE) [30], fewer than half of patients with eating disorders recover and 20% develop longstanding eating disorders (L-ED) [22, 44]. This patient group is often complex with extended treatment histories, multiple hospital admissions, a poor quality of life and the highest mortality rate of all eating disorders [28, 41, 48].

There is a lack of agreement amongst clinicians about how to define L-ED [9, 47, 56] and attempts to use statistical modelling to differentiate this group have been unsuccessful [54]. Several researchers have attempted to define or describe the group of patients experiencing L-ED by using the concept of a ‘severe and enduring eating disorder’ (SEED) [39]. A ‘Staging Model’ used illness duration as criteria suggesting that eating disorders lasting for 7 years or longer would be defined as SEED [10, 50, 51]. Broomfield et al. [9] however, pointed out that patients can have L-ED but not be ‘severe’ and vice versa. Similarly, Marzola et al. [27] found that ‘enduringness’ was not a specifier for severity or outcomes between long, medium and short duration inpatients with anorexia nervosa. The lack of clear agreement about how to define and describe this group of patients presents challenges for clinicians and researchers to understand how to best support them.

There is little research into how to best support patients with L-ED and in the UK the NICE [30] guidelines offer few specific recommendations. Australia and New Zealand offer the most comprehensive guidelines with clear recommendations including maintaining hope, taking a harm minimisation approach and improving quality of life [20]. Likewise, the American Psychiatric Association (APA) guidelines encourage hope but also suggest the possibility of moving away from traditional eating disorder treatments [60]. This patient group are often complex with extended treatment histories and multiple hospital admissions as well as the highest mortality rate of all eating disorders and a poor quality of life [28, 41, 48]. When working with those with L-ED there continues to be tension between whether weight restoration and reducing eating disorder symptoms should remain the primary goal or whether there can be a legitimate focus on harm reduction and improving quality of life at certain stages of care [42]. A review summarising the limited literature suggested treatment taking a multidisciplinary approach and focusing on creating a collaborative therapeutic relationship to establish appropriate treatment goals, which may not involve reducing eating disorder symptoms [57]. However, there is a growing body of research suggesting that evidence-based treatments can lead to recovery in those with L-ED [5, 12, 27]. For instance, a randomised controlled trial found that existing evidence-based treatments result in significant improvements amongst patients with L-ED, and are acceptable to this population with only a 15% drop-out rate [49]. The lack of agreement on how to define and support patients with L-ED means that care is often inconsistent between services.

Although the evidence suggests that existing evidence-based therapies can lead to change in those with L-ED, it is also important that services are flexible and responsive to the needs and goals of this group of patients at all stages of their treatment journey in order to maintain engagement, hope, and to enable co-produced, personalised care planning. Adapting existing treatment pathways to incorporate this way of working will require changes in staffing levels, training, skill mix and redesigning of care pathways. Given that supporting this group of service users can be resource intensive [56], it is important that resources are used in the most effective way. Therefore, this project set out to explore how to best define, describe and support patients with L-ED by inviting and integrating views of service-users, staff, and the health guidance and research literature.

### Theoretical basis of project

There is no single evidence-based theory of L-ED, however, parallels can be drawn with chronic health theories such as the Chronic Illness Theory (CIT) [29]. CIT makes several predictions about individual and environmental factors which contribute to adjustment such as high self-efficacy, acceptance, social support and treatment adherence. Although there is no known research testing CIT in relation to mental health disorders, it has face validity for L-ED and has previously informed interventions to support adjustment to chronic physical health conditions such as spinal cord injury, diabetes and kidney disease [13, 15, 24]. This theory informed the design, analysis and interpretation of the project as authors sought to establish whether the same factors contributing to successful adjustment are also present in L-ED.

### The current service

This project took place in the UK whereby the National Health Service (NHS) is split into several Trusts, each responsible for providing care to different areas of the UK. Within the NHS Trust this project took place in, the eating disorder services consist of a community child and adolescent eating disorder service (CAEDS) for those up to 18 years of age, an adult community eating disorder service (A-ED) for those aged 18 and over and specialist adult inpatient unit for patients requiring more intensive treatment. In CAEDS, individuals with L-ED who have not recovered following standard evidence-based treatments are offered a formulation-based personalised care package, and many subsequently transition to A-ED. In one area, A-ED offers a support and monitoring pathway to those with L-ED who are not currently engaged in evidence based treatment. This pathway predominantly monitors patients’ physical health alongside goal-directed support based on the individual’s formulation and personalised goals. In the other area, there is no designated pathway, instead a nurse consultant takes the lead for patients with L-ED and offers consultation to care coordinators.

Anecdotally staff report that supporting these patients requires a large amount of staff time and resources due to their complex needs which do not fit with the existing evidence-based approaches offered within the service. Therefore, in line with the NHS long-term plan, the service wished to involve patients in service development and was keen to learn from their experiences to improve the provision of person-centred care and ensure that support for this group is appropriately resourced in the most effective way [32].

### Aims of the project

In order to offer those with L-ED more consistent, person-centred and evidence-based care, the service required recommendations to inform the development of a lifespan pathway across eating disorder services. Therefore, to identify who may benefit from the new pathway, this project aimed to evaluate the most helpful way to define and describe individuals with L-ED. Additionally, it aimed to synthesize research evidence, clinical experience, and patient experience to understand how to best design a new pathway to meet the needs of such individuals. Specifically, the project addressed the following:

1. What is the best way for the service to identify those with L-ED?
2. What proportion of the current NHS A-ED and CAEDS caseload have L-ED?
3. How can the NHS eating disorder service develop a care pathway which better meets the needs of service-users with L-ED?

## Methods

### Design

This service improvement project utilised a mixed-methods design over three stages which are detailed below. The methodology and interview schedule were designed in collaboration with patients with L-ED. One dedicated staff member from each service was closely involved in the design and delivery of the project to ensure the impact on staff was limited.

### Ethics

The project was approved by the local Research and Development Team. All eligible service-users and staff were provided with an information sheet and consent form which contained details about the project and the researcher’s email address to allow them to ask any questions. All participants provided written informed consent before taking part in interviews or focus groups. Participants were guided to make a unique participant identifier which was used to store data anonymously and to allow participants to withdraw their data. Participants could also indicate if they wished to receive a summary of the results of the project; the email addresses of those that did were stored separately from their data.

### Procedure

#### Stage 1: literature review

A literature search was conducted to answer the first question by reviewing the existing literature on defining and describing those with L-ED. The search was conducted between July and September 2020 using the following databases: PsycINFO; Web of Science; Medline; Embase; CINAHL and reference lists of relevant papers. If necessary information was missing from the full text, or was unavailable, first authors were contacted via email. The search terms were as follows: (chronic OR longstanding OR severe enduring) AND (eating disorder OR anorexia OR bulimia) AND (defin* OR treat* OR understand*).

**Fig. 1:**
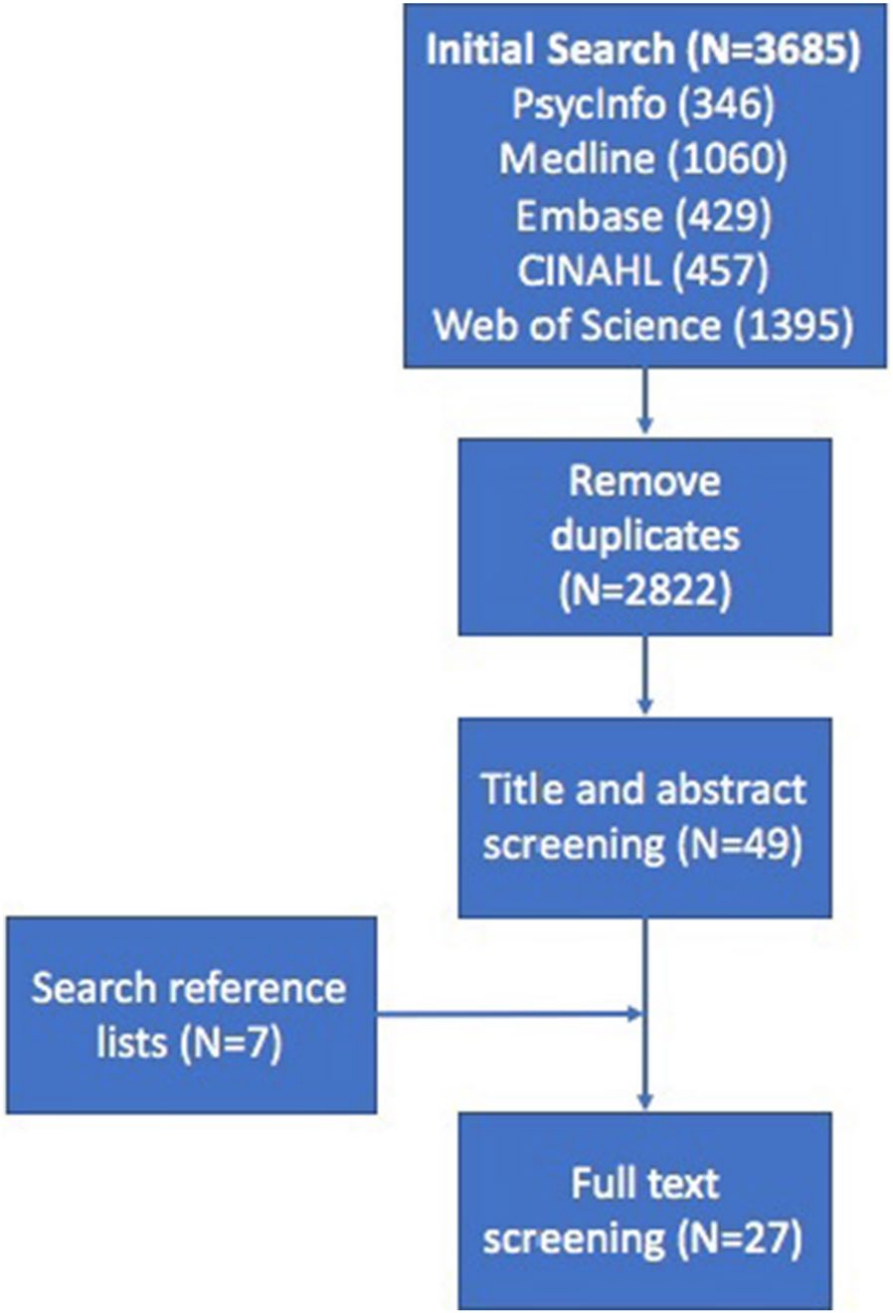
Flow chart detailing the number of papers included at each stage of the literature review

Papers were included if they were a journal article published in English which provided a definition of L-ED. Reviews were not included unless they provided a definition, or criteria for L-ED in their results. Due to the relatively small body of literature on L-ED, all other relevant papers were included regardless of their methodology. A flow chart detailing which papers were included at each stage is seen in Fig. 1.

A data extraction form was used to gather key information from relevant papers, see Additional file 1. This was used to draw comparisons and extract common criteria and terminology which was then amalgamated to create a provisional definition of L-ED, this is described below in the results section. This definition was used to identify suitable participants for stage two of the project and was discussed during interviews and focus groups.

#### Stage 2: qualitative data

The second stage of the project involved conducting staff focus groups and individual interviews with service-users with L-ED. All service-users who met the provisional criteria from stage one were approached by their care coordinator about participating. There were 21 interested service-users who were provided with the information sheet and consent form. Of these, 12 indicated they would like to proceed and were contacted by researchers to arrange an interview. Reasons for not taking part included a deterioration in physical health, managing complex social situations and not wishing to take part at the time interviews were being conducted.

All staff working in local eating disorder services received the information sheet and invitation to take part in focus groups via email and during team meetings. Twenty eight interested staff completed the consent form before attending one of three focus groups which took place via Microsoft Teams. CAEDS staff, A-ED staff and management staff were separated to allow for open discussions. All interviews and focus groups were conducted by the main researcher. To reduce the impact of bias, the researcher completed a bracketing interview prior to the first interview where they discussed their hopes, expectations and concerns about the project, they also kept a record of observations and biases throughout recruitment and analysis.

Interviews and focus groups followed a semi-structured interview schedule. These took place in person, on the phone or via video call based on patient preference and NHS social-distancing guidelines which were in place throughout recruitment due to the Covid-19 pandemic. All interviews and focus groups were audio recorded and transcribed.

#### Stage 3: quantitative data

The final stage involved gathering information about how many service-users would meet the L-ED criteria. The results of stage one and two were combined to create a final definition of L-ED. This was distributed to staff teams who reported to researchers how many service-users on their caseload met the criteria. Researchers then combined this information to understand how many service-users in each team have L-ED.

### Participants

Demographic information about participants can be found in Tables 1 and 2. Twelve service-users took part in interviews. Amongst all service-users, the mean length of eating disorder was 19.6 years. Two participants were recruited from CAEDS who each had an eating disorder duration of 4 years. The ten adult participants had a mean eating disorder duration of 22.7 years. Twenty-eight staff took part in focus groups, the majority of staff were female, and they covered a wide range of professions across the services.

**Table 1:** Demographic information regarding the service-users who took part in interviews

Demographic variable	Mean (M) or number of participants (N = 12) [standard deviation] (percentage)	Range (years)
Age	M = 34.4 years [15.3]	17–68
Ethnicity	White British N = 12 (100%)	
Gender	Female N = 12 (100%) Male N = 0 (0%)	
Length of eating disorder	M = 19.6 years [14.5]	4–51
Patient status	In patient N = 5 (42%) Outpatient N = 7 (58%)	
Service	A-ED = 10 (83%) CAEDS = 2 (17%)

**Table 2:** Demographic information regarding the staff who took part in focus groups

Demographic variable	Mean or number of participants (N = 28) (percentage)
Gender	Female N = 24 (86%)
	Male N = 4 (14%)
Focus group type	A-ED = 10 (36%)
	CAEDS = 7 (25%)
	Leadership = 11 (39%)
Job role	Administrator N = 1 (3.6%)
	Assistant psychologist N = 2 (7.1%)
	Clinical psychologist N = 5 (17.9%)
	Counselling psychologist N = 1 (3.6%)
	Dietician N = 4 (14.3%)
	Nurse N = 3 (10.7%)
	Psychiatry N = 4 (14.3%)
	Senior mental health practitioner N = 1(3.6%)
	Social worker N = 1 (3.6%)
	Support worker N = 1 (3.6%)
	Systemic family therapist N = 2 (7.1%)
	Team manager N = 2 (7.1%)
	Trainee clinical psychologist N = 1 (3.6%)

### Data analysis

Quantitative data was collected using Microsoft Excel. The qualitative data was analysed using the Framework Analysis protocol [38]. Three researchers coded the first transcript independently before meeting to compare initial codes and create a shared language for codes, any disagreements were resolved during discussions. Two further transcripts were then coded independently, and researchers met again to generate and agree on the codes which were included in the final analytical framework. The analytical framework included a mixture of inductive and deductive codes based on CIT [29]. This analytical framework was then applied to the other transcripts,data saturation was reached as no new codes were added from the later transcripts. Researchers continued to meet regularly throughout the process to carry out reliability and validity checks by discussing biases, discrepancies and ideas for themes. As per the framework analysis protocol, a framework matrix was created to support researchers in interpreting the data and identifying themes.

## Results

### Stage 1: literature review

The results of the literature review identified twenty-seven relevant papers which are summarised in a data extraction form in the Additional file 1. In response to the first research question, data on how to define and describe L-ED was extracted from each paper and is summarised below.

The majority of papers in the literature review used a duration criterion which ranged from 3 to 20 years (e.g. [12, 33, 41, 46﻿]). The most common was 7 years, however, several reported this was unhelpful and arbitrary (e.g. [47, 54]). Other common criteria included previous evidence-based treatments which have not worked (e.g. [7, 16, 21, 59]), persistent symptoms (e.g. [47, 61]), requiring monitoring by a health professional (e.g. [40, 53]); a low BMI (e.g. [10, 23]), and low motivation to recover (e.g. [7, 18]).

The results of the literature review revealed that the majority of articles use the SEED concept or a variant of this (e.g. [2, 12, 36]). However, five of these papers reported results from one randomised control trial from a research group which used “SEED” to describe a L-ED [1, 6, 25, 45, 49]. Other labels included “chronic” (e.g. [14, 16]), “long-term” [18] and “longstanding” (e.g. [7, 10, 11]).

This data was summarised into the below provisional definition of L-ED which was used in stage two:

Persistent Symptoms

- A functional impairment
- Require regular MDT monitoring
- Shape and weight cognitions
- Dietary restraint behaviors
- Underweight (BMI < 18.5 as per DSM-5 and WHO criteria)

Enduring

- 7 or more years

Treatment Resistance

- Previous evidence-based treatment which has not worked.
- Low motivation to recover

### Stage 2: interviews and focus groups

**Fig. 2:**
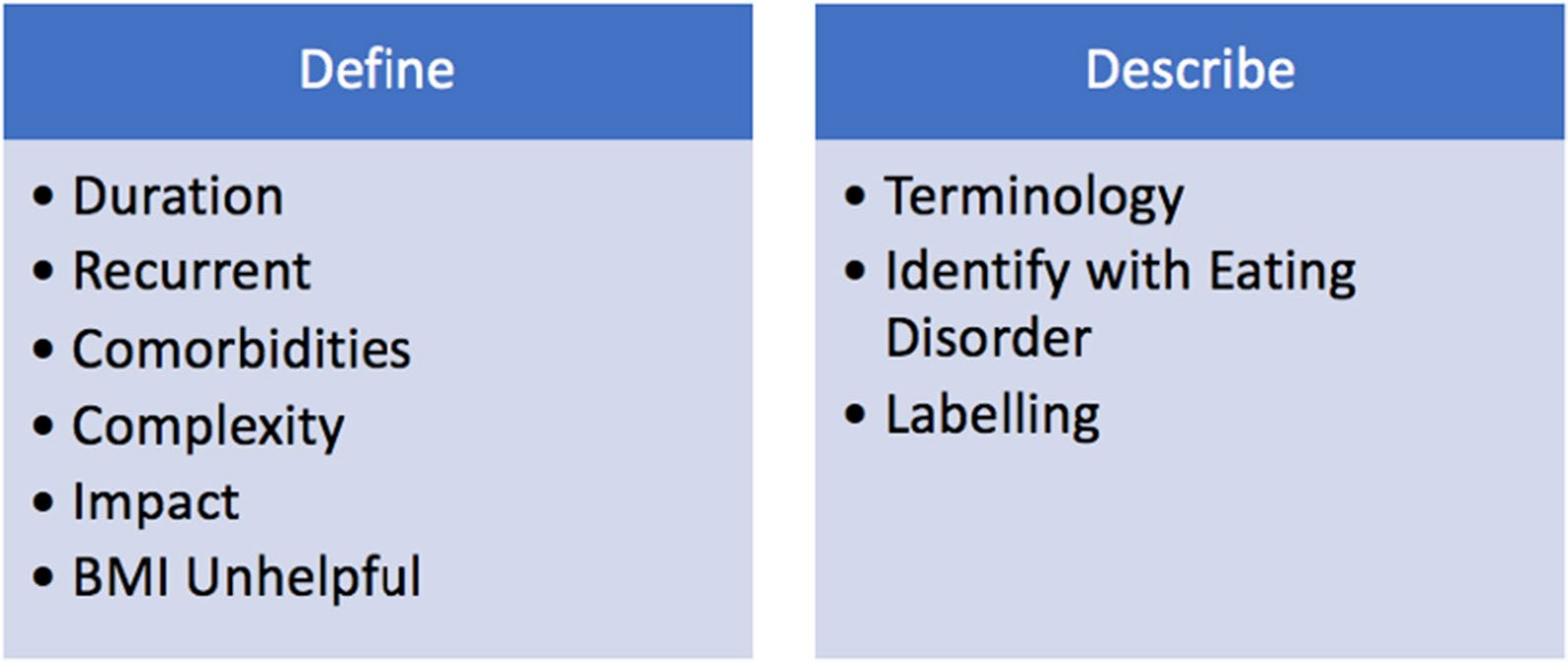
Map of themes in relation to research question one: what is the best way for the service to identify those with L-ED?

**Fig. 3:**
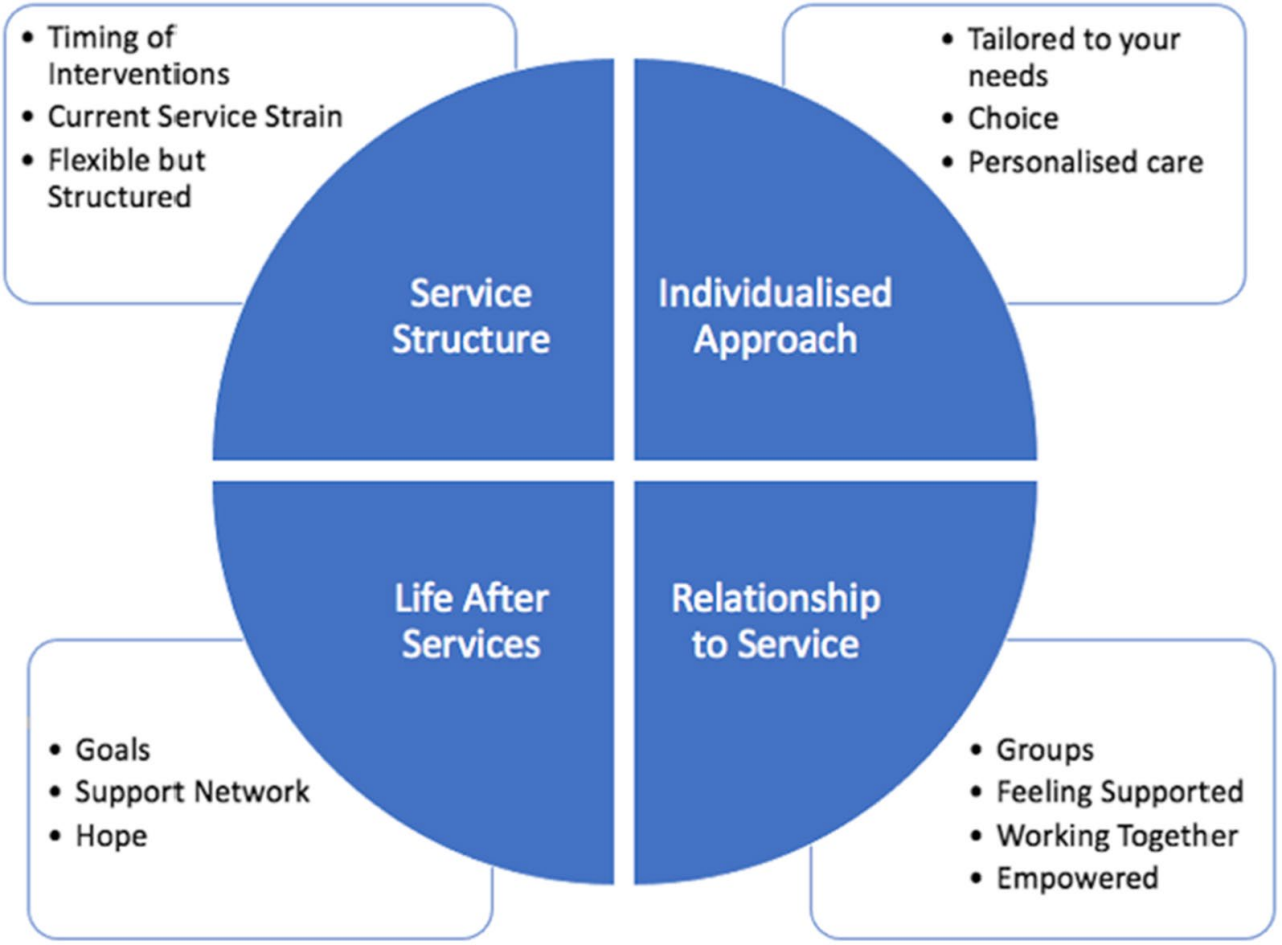
Map of themes and subthemes in response to research question three: how can the NHS eating disorder service develop a care pathway which better meets the needs of service-users with L-ED?

The results of the interviews with 12 service users and focus groups with 28 staff were analysed into six broad themes which were each divided into subthemes, these are depicted in Figs. 2 and 3. Additional illustrative quotes for each theme are available on request.

#### What is the best way for the service to identify individuals with L-ED?

The qualitative data was initially used to answer the first research question. Framework analysis identified two main themes in relation to question one labelled ‘define’ and ‘describe’. These are described below.

##### Define

The ‘define’ theme was divided into six subthemes to represent the six features that staff and service-users felt to be important in differentiating L-ED. In line with the research literature, the most common feature was duration. There was a sense that the eating disorder had become lifelong, although some agreed that a specific cut off was “*arbitrary*” [staff, CH23].

Eleven service users also reported on the recurrent nature of L-ED whereby relapse is common and recovery short-lived, this was also raised in all staff focus groups. This was particularly evident when discussing inpatient admissions, with service-users describing the process of being hospitalised and discharged as a “*vicious cycle*” [service-user, GT73] which “*just repeats itself*” [service-user, BR13]. There was a feeling that this cycle was futile, and a new approach was needed to break the pattern.

*“if you’ve done the same thing like four or five times and it hasn't worked it seems a bit insane to keep going with it,” *[service-user, HE17]

Additionally, they described the eating disorder becoming more complex and frequently including physical or mental health comorbidities *as “over the years, the condition gets more complicated, because other problems come along”* [service-user, HT84]. Participants reported that the impact of illness amongst those with L-ED was also great. They reported persistent, serious symptoms which had a large impact on their day to day functioning as well as a sense that their *“life has been lost”* [service-user, RI30].

*“I think once you’ve had an eating disorder for a certain amount of time… you can't just have just an eating disorder because it affects so many things of your life that, you’re more - whatever is wrong with you becomes more than that, so you also have depression and stuff like that, so anxiety you know your self-esteem goes down. So, you know if it wasn't bad at the start, it's a lot worse at the end.”* [service-user, RI30]

A clear message from the majority of participants was that including a BMI cut off in the criteria would be unhelpful. One service-user commented on the validity of BMI “*comparing you to mere statistics*” [service-user, BR13]. Adult and leadership staff also expressed concerns about a BMI criteria and eight service-users leant on personal experiences such as being denied help if their BMI reached a certain level and simultaneously struggling more as their BMI increased.

##### Describe

Despite the literature frequently using “SEED”, the majority of service-users and staff expressed a dislike for this term. Eight service-users reported a preference for longstanding over chronic, as did several staff members.

*“…because longstanding kind of seems like it's been a long time, but it doesn’t have to always be that way, whereas chronic seems to imply that it's just gonna be there forever”* [service-user, SM31].

However, nine service-users discussed the pitfalls of using any label at all with one service-user stating “*you need to scrap that completely”* [service-user, BR13]. They reflected that their eating disorder had become intertwined with their identity and were concerned that an additional label or diagnosis could negatively impact on the care and treatment they receive.

*“I’m seeing up and down in the country is that the SEED patients don't get active treatment, they don't get admitted to hospital for weight restoration but they kind of go around in revolving door admission and discharge”* [staff, JQ14]

#### How can the service develop a care pathway which better meets the needs of service-users with L-ED?

The results of the interviews and focus groups were then used to answer research question three, data was analysed into four themes which are described below.

##### Service structure

One theme described how the new pathway should be structured. The first subtheme highlighted the importance of matching the timing of psychological interventions to service-users’ readiness to change as opposed to their level of physical risk. Service-users described support not being available at the right time with many citing “*a massive gap*” [service-user, HE12] between in and outpatient services. Some felt there were barriers to accessing support in the community when they have looked for it and felt that it was now too late for them.

The second subtheme highlighted the impact of NHS financial pressures on the current service structure, with one service-user stating “*I’m not sure that they’re meeting those needs [of those with L-ED], or that they feel they’re able to*” [service-user, LE27]. Others drew comparisons to chronic illnesses such as schizophrenia or diabetes and called for L-ED services to be commissioned similarly to offer long-term, flexible support.

*“Whereas say if it was someone with like schizophrenia you’d really tamper with their medicine, you’d do all sorts of things so that they could be sustainable in, you know, in society. You’d, you know if it was someone with bipolar disorder you wouldn’t just cast them aside and say ‘we tried all the things, so we’ll just cast them aside’” *[service-user, RI30]

It was clear that the new pathway should offer “*greater flexibility*” [staff, ST63] and some cited the introduction of remote working during Covid-19 as a successful example. However, others felt that providing structured support was important, such as utilising a *“CTO and… [the] parameters of that”* [service-user, HE17] or having a clinic with clearly defined roles and expectations.

*“I can see there is a value to having a structured clinic so that there is different personnel in that clinic who can take on different roles, or different, you know parts of managing the different needs of the patient.”* [staff, LE31]

##### Individualised approach

The second theme describes the need for *“an individualised approach”* [MA14]. The first subtheme relates to treatment being tailored to service-users by offering access to a range of professionals, as “*having a multidisciplinary team is really important*” [staff, JQ14]. The provision of psychological therapy must also be individualised as some felt it should be readily available “*regardless of their weight*” [service-user, LE27] whilst others did not want *“a continuous push for psychological interventions”* [service-user, HE17]. The importance of having physical health checks to manage the risks of L-ED was clear, although this should be needs-led as some described “*too much emphasis on the weight*” [service-user, FA26] and a desire to “*eventually move away from doing it every week*” [service-user, HE12] as they felt this negatively impacts on their wellbeing.

The second subtheme described the importance of patient choice. Many felt that currently they were passive receivers of care and that they “*have no control”* [service-user, HT84]. Therefore, staff and service-users were aware that it would be key to a new pathway that “*people should be given more choices*” [service-user, GT73] and control over their care.

*“just listening to your reasons why. Like if an admission isn’t gonna be helpful then like understanding why it’s not going to be helpful and what they can do instead”* [service-user, LP02]

The final subtheme summarised the importance of offering personalised support. One example was ensuring that treatment is age appropriate as older service-users reported feeling *“as though they [staff] were just dealing with sort of run of the mill, 18–25-year-olds that have got an eating disorder*” [service-user, PA11]. Others spoke about looking beyond the evidence base and instead tailoring treatment based on a bespoke formulation.

*“I would probably think about it in terms of someone who's exhausted, the kind of options of evidence-based treatment and probably need something a little bit more tailored.” *[staff, LI08]

##### Relationship to service

The next theme describes how service-users relate to the service. The first subtheme describes how service-users could build supportive relationships with each other. Many felt that peer-support groups would be beneficial, and staff suggested utilising technology to access groups remotely. Some proposed peer-support workers facilitating groups as service-users found it “*inspiring*” [service-user, SM31] to speak to recovered service-users.

*“I think it would be very helpful… if you had had a sort of regular support group for people who you know were long-term sufferers. Or, and also for people who had recovered after a long-term, so you'd have that element of hope in there as well”* [service-user, HT84]

The second subtheme highlights the importance of *“building up trust with somebody*” [service-user, HT84] from the team. This requires staff to provide consistent, responsive care and “*continuity*” [service-user SM31] to allow service-users to feel safe and supported.

“*they tend to be really quite distrusting people so really that engagement and continuity is really quite important.”* [staff, JQ14]

The working together subtheme highlights the importance of service-users, staff, and other professionals providing joined-up care. Service-users described feeling blamed for not recovering and would like increased information to allow greater collaboration “*to work with them [staff] instead of against them”* [service-user, HE12]. Due to the complex nature of L-ED, many service-users are supported by multiple health teams and it was felt that inter-agency working, and communication required improvement as currently “*there isn’t really a connect between the two*” [service-user, LE27].

*“So, that is all just connecting the dots, except that breast cancer was separate from the eating disorder but the trouble is, you’re all one person*.” [service-user, PA11]

The final subtheme describes empowering service-users to change their relationship to their eating disorder by becoming more independent and “*taking the reins*” [service-user, GT73]. Several felt the pathway should build service-user’s confidence and self-efficacy in their abilities or encourage them to access community services which are separate from NHS services.

##### Life after services

The final theme describes preparing patients for life after services. The first subtheme relates to the aims and goals of the pathway. Service-users felt that currently the service defined recovery as weight-restoration with and stated that “*if I’m not up for full recovery then I’m kind of not their business*” [service-user, LE27]. Many felt the pathway should instead focus on learning to *“manage and maintain”* [service-user, GT73] weight and improve quality of life. However, some staff had concerns about how this approach could be managed safely.

*“diseases that ruin life don’t work with quality of life”* [staff, CH23].

*“…making your life have meaning, building connections and- so that the, you know, even if you can't recover completely, so that your life had, had more meaning you had more, you know, was richer”* [service-user, HT84]

The second subtheme describes the value of building a support network outside of eating disorder services. Many spoke of “*[losing] all my friends in the depths of eating disorder”* [service-user, FA26] as “*it is a very isolating disease”* [service-user, GT73]. Staff also described L-ED resulting in strained relationships and burnout as “*it can be emotionally very draining”* [staff, JQ14]. As a result, service-users sought support from social media, although many recognised that this was not always helpful, and support was required to navigate this. Therefore, the need to offer support to build helpful connections outside of mental health services was clear.

The final subtheme highlights the importance of providing hope. Many felt that the current service could feel hopeless as though “*they just give up on you*” [service-user, SM31].

*“they kind of told me a couple years ago, even like, I was probably there for about a year and a half and they told me, they, they'd done everything that they could at that point” *[service-user, FA26]

Therefore, the new pathway should promote a hopeful message and *“talk of recovery… that this can get better”* [service-user, GT73].

*“I guess to hold some hope that that, that is possible, and I guess, I imagine many of us have had experience with people with very long-term illness that had actually at some point changed and made some massive steps towards full recovery in many cases. So, I guess holding on to that hope is really important."* [staff, CH23]

These themes informed a series of recommendations which can be found below, to support the service to better meet the needs of those with L-ED.

### Stage 3: quantitative data

Finally, the results from stages 1 and 2 were combined to create a proposed definition of L-ED. This was used to answer research question 2 by identifying how many service-users in each team have a L-ED. The proposed definition was as follows:

“Three years active treatment (can be multiple episodes of care which add up to 3 years) with severe symptoms (cannot be discharged) which have a significant impact on day-to-day functioning (e.g., disruption to education or employment; significant time spent in hospital; social disruption).”

- May also have comorbidities, several hospital admissions and a low BMI, but these are not necessary to meet the criteria.
- Should have all been offered at least one evidence-based treatment which they have not responded to or engaged with.

The data revealed that a total of 109 service-users met the proposed L-ED criteria. This consisted of 17 (5%) of the 340 service-users on the CAEDS caseload and 92 (21%) out of 436 service-users on the A-ED caseload. This data describes how many service-users meet the criteria for the pathway, as opposed to those who have expressed an interest in engaging with the pathway.

## Discussion

### Clinical recommendations

This project identified a clear need for a dedicated pathway to support service-users who meet the L-ED criteria which offers structured, specialist, lifespan support. The results identified that this pathway would support service-users who continue to experience severe eating disorder symptoms which have a significant impact on their functioning, despite at least 3 years of previous active treatment. Crucially, the results identified that the pathway may use the term L-ED to describe the pathway however, inclusion in the pathway should not result in any label being applied to service-users (such as SEED).

Recommendations for the implementation of the pathway are detailed below:

Goals of the Pathway: 

- Main priority to improve quality of life and achieve physical stability.
- Care is person-centred as service-user’s preferences are heard, respected and used to design an individualised, formulation-led care plan.
- Promotes a hopeful message about recovery but this is not solely defined as symptom reduction, could alternatively be defined as accessing employment, relationships or education.
- Service-users who are physically compromised to be informed that recovery involves improving physical health status, including weight restoration. However, should they decline to engage with weight restoration, legal frameworks such as The Mental Capacity Act and Mental Health Act should be used to assess their capacity to make unwise decisions and manage the health risks associated with this.
- Accessing the pathway should not prevent service-users from accessing recovery focused admissions or treatments in the future should they wish to do so.

Structure of the pathway:

- An appropriately staffed multidisciplinary team to allow service-users to access tailored support that suits their needs.
- Clear expectations and structured support to ensure service-users can make an informed choice about joining the pathway.
- Clear timeframes for reviewing service-users’ engagement and progress with the pathway using appropriate goals based outcome measures. As in chronic physical health settings, service-users can leave and re-join the pathway in the future as needed.
- Support delivered flexibly by offering support virtually and outside of office hours.
- Service-users to have a consistent key worker, or team of professionals who they are in regular contact with, including email and phone support.
- To consider a non-crisis phone line that service-users can call if they require additional support outside of their allocated appointments.
- Regular physical monitoring by the service or GP with a clear rationale for the frequency of monitoring and good communication between professionals and service-users.
- The option to access NICE recommended therapy.
- Offer a range of peer support groups with involvement from recovered service-users.

Linking the Pathway to Existing Services:

- To build on the existing ‘whole of care’ pathway model to offer intensive community support which aims to maintain stable health status, as well as providing an alternative to, or support following, discharge from an inpatient admission.
- To explore the possibility of technology enabling service-users to attend treatment groups regardless of inpatient status.
- To consider specialist staff from the pathway offering consultation to other staff, including other health teams and during transition from CAEDS to A-ED services.
- To build links with non-NHS community services to encourage service-users to become autonomous.

### Relationship to existing literature

The results add to the literature on defining, describing and supporting those with L-ED. Interviews with service-users and focus groups with clinicians within this NHS service identified that several participants expressed a dislike for an “arbitrary” 7 year cut off, the majority suggested removing BMI as a criterion, and almost all participants highlighted a preference for the term L-ED over SEED.

The project also identified that approximately 5% (CAEDS) to 20% (A-ED) of service-users could benefit from a treatment pathway specifically tailored to the needs of patients with L-ED. This is consistent with other research into the prevalence of L-ED [44]. It is important to bear in mind that these figures relate to the number of service-users who are eligible for the pathway, as opposed to how many wish to access it. A proportion may choose to pursue evidence based, recovery-focused treatments which include weight restoration, and they should be supported to do so as these remain effective in this population [12, 14, 49]. For other patients who do not currently wish to access recovery focused treatments, this research supports the recommendations from the NHS Long-Term Plan [32] which highlighted that person-centred care, peer support and patient choice are integral to supporting those with severe mental illness.

This project highlighted disagreement as to whether full weight restoration should always be a primary goal in L-ED services. Management staff viewed weight-restoration as a “key goal” of treatment [30] and cited evidence that full recovery remains possible in those with L-ED who opt for weight-restoration [37]. On the other hand, some clinicians and service-users wished to choose to focus on stabilisation, maintenance and improving quality of life. This approach is in line with the Australian and New Zealand treatment guidance [20] which is supported by research [53, 56] and has been successfully implemented in other NHS services [43]. CIT describes both safe medical monitoring and perceived control over illness management as important [29], however a different approach may be warranted for L-ED as this project highlighted that these two elements may not always be compatible and some may consider supporting patients to remain at an unhealthy weight to be ‘colluding’ with the eating disorder.

Adopting Prochaska and DiClemente’s [34] Stages of Change Model could resolve this dilemma by matching the intervention with service-user’s readiness to change. The results of this project and CIT [29] highlight that focusing on self-efficacy, social support, and good medical behaviours could reduce distress for those not yet ready to fully recover. Evidence shows that this type of work to increase motivation predicts better treatment outcomes in future recovery-focussed interventions [17, 26]. The pathway should always share that recovery remains possible in L-ED [35] to further motivate service-users to move to the preparation and action stages of change. Matching the intervention to service-users’ readiness to change promotes a hopeful message as the community team can offer interventions to reduce distress, even if service-users are not yet ready to pursue full recovery.

As indicated above, this project provided evidence that CIT [29] applies to L-ED, as CIT predicted many of the subthemes identified in the analysis. However, whilst CIT acknowledges the role of social support and professional relationships, it predominantly focuses on individual factors, whereas many of the subthemes identified in this project were relational. The benefits of peer-support for this population were clear and are supported by research that peer-mentorship provides hope and motivation, as well as allowing service-users to re-engage with the world [19]. The NHS long-term plan also specifically states that eating disorder services must develop their peer support workforce and offer more group support [32]. Therefore, although CIT remains a helpful overall framework for the pathway, it is important to also consider relational factors.

### Strengths and limitations

A strength of this project was that patient experience was at the heart of service development as service-users could shape how they are described and supported. For example, the majority of service-users disliked being labelled as “SEED”, despite this term frequently being used in the academic literature. Their concerns are in line with other clinicians [47] and patients with other chronic mental illnesses who report that labelling negatively impacts public attitudes towards them and serves as a barrier to treatment or employment [3, 52].

However, the sample size in this project was small and all service-users were White British females which makes it unclear if their views represent others with L-ED. Additionally, as data was not collected anonymously, some staff or service-users may have felt unable to take part or speak freely during interviews and focus groups. Despite these limitations, the project reached data saturation which suggests that the sample was adequate to represent the views of those within the NHS trust.

### Impact and dissemination

All participants who requested a copy of the results were sent a summary. The results were also shared with the clinical and operational leads for eating disorder services within the trust to guide service transformation including workforce planning to develop a consistent pathway across teams for L-ED which could support up to 20% of the caseload. Additionally, the recommendations were presented directly to staff in each team, as well as in the Trust-wide monthly development slot.

## Conclusions

This project highlighted some of the challenges and controversies that arise when attempting to define and describe those with L-ED. We found that the majority of patients and staff prefer to use the term L-ED compared to SEED or chronic eating disorder, and dislike the use of arbitrary duration cut offs or BMI criterion. We propose that some patients with L-ED who are not yet ready to work towards traditional recovery may benefit from an alternative pathway which focuses on improving quality of life through person centred support. However, the results also emphasized the importance of hope for those with L-ED, including supporting them to access recovery focused treatments should they wish to do so.

This project was specific to one NHS trust in the UK therefore future research must establish if the recommendations generalise across other settings. It is also important for future research to analyse the efficacy and acceptability of the proposed pathway. Research should consider the use of values-based outcomes which matter to service-users such as quality of life, increased self-efficacy, improved relationships and reduced hospitalisations, whilst also establishing its cost effectiveness. Importantly, clinicians and academics should listen to the service-users’ preferences and consider changing their language and criteria to better reflect service-user experience.

## Supplementary Information

**Additional file 1.** Data extraction form. This is the data extraction form used during the literature review. The data extraction form was used to extract the language and criteria used in each of the identified papers.

## Data Availability

The datasets used and/or analysed during the current study are available from the corresponding author on reasonable request.
