# Supplementary material for: Creating a care pathway for patients with longstanding, complex eating disorders
Source: J Eat Disord. 2022 Aug 29;10:128. doi: 10.1186/s40337-022-00648-0 (PMC9421634; doi:10.1186/s40337-022-00648-0)
Supplement: Supplementary file 1 — Additional file 1. Data extraction form. This is the data extraction form used during the literature review. The data extraction form was used to extract the language and criteria used in each of the identified papers. [file 40337_2022_648_MOESM1_ESM.pdf]

Author and year	Country	Participants	Terminology used	Criteria for longstanding Eating disorder	Methodology	Results
Abd-Elbaky et al. (2014)	Australia and UK	63 with SE-AN	Severe and enduring anorexia nervosa (SE-AN)	≥ 7 years	Part of larger RCT. 30 sessions CBT or SSCM. Looked at stages of change and illness subtypes in SE-AN	Purging type sig more likely to drop out. Poor QoL also predicts drop out. No differences between type of therapy
Ambwani et al. (2019)	UK – SHARED trial	187 patients from 22 eating disorder services	Severe and enduring anorexia nervosa (SE-AN)	More info in supplementary material Severe psychological distress (DASS>60) AND duration ≥ 7 years	Secondary analysis to compare outcomes in SE-AN with early stage AN at postintervention, 6m and 12m	SE-AN more likely to have used inpatient or day patient care (82.9%), worse EDE-Q and WSAS scores than early stage
Bamford & Mountford (2012)	UK	n/a	Longstanding anorexia nervosa (L-AN)	Consistently ill for ≥10 years (short periods of weight restoration 6m or less included), at least one recognised therapeutic treatment in this time, show severe impairment across several life domains, express low motivation to recover	n/a	Describes how to adapt CBT for L-AN
Bamford et al., (2015)	UK	63 female's diagnosis of SE-AN	Severe and enduring anorexia nervosa (SE-AN)	≥7 years	RCT, 30 sessions or either CBT-AN or SSCM (specialist support and clinical management), measures baseline, 15 weeks, end of treatment, 6, 12-month f/u. EDE, EDQOL, SF-12, WSAS, BDI	BMI significantly related to future quality of life (QoL). EDE sig associated future QoL. In SE-AN should focus on QoL, but also need to improve BMI and ED symptoms
Broomfield et al., (2017)	Australia	32 studies in systematic review, 9	Label in order of prevalence: chronic, SEED,	84% use duration of illness, usually 7 years.	Systematic review to try to define criteria for SE-AN	Feel chronic shouldn't be used as implies in-curable, prefer SEED

		RCT, 23 non-RCT	longstanding, critical, refractory, prolonged, treatment-refractory, treatment resistant, persistent	41% use previous failed treatment<br>6 articles use BMI below 17.5<br>3 articles say pattens related to behaviour + cognition "entrenched patterns of food restriction"		
Calugi et al., (2013)	Italy	95 women Consecutively underweight admitted to ED ward. Failure of previous treatment, no substance mis-use.	Longstanding eating disorder (L-ED)	≥ 10 years	Adapted CBT-E for inpatient setting (CBT-I). Compared longstanding with not longstanding	All L-ED pts improve BMI by end of treatment but no significant differences in recovery between groups
Calugi et al., (2017)	Italy	66 women with AN. Same criteria as above for admission	Severe and enduring anorexia nervosa (SE-AN)	> 7 years	Compared outcomes of CBT-E SE-AN vs non-SE-AN in an inpatient unit	Found improvements in BMI in both groups and at 12 months follow up: disagree that need to shift to focus on QoL in SE-AN
Dawson et al., (2014b)	Australia	8 women had AN for > 7 years but now recovered for ≥ 5 years	Chronic AN (C-AN)	> 7 years	Interviewed recovered pts about their experience of recovery, used narrative enquiry.	Create framework to understand how those with C-AN recovery. 4 phases of recovery 1)unready, 2) tipping point, 3) active pursuit recovery, 4) reflection and rehabilitation

Fox & Diab, (2015)	UK	6 patients in ED ward due to low BMI or risk to self	Chronic AN	≥ 6 years (all also had BMI 14/15; at least two previous unsuccessful therapies and had prior admissions, but not a requirement)	Interviews and IPA	Themes: making sense of AN; experience of treatment; interpersonal relationships; battling with AN; staff pessimism
George et al., (2004)	Australia	8 patients	Long-term/ chronic AN	≥ 7 years which had not improved following previous treatment. Refusing to engage in 'active' treatment.	Examine motivational enhancement therapy intervention with long-term AN	Pts showed increases in motivation to change after 6 months, found it helpful that no expectation of behaviour change, just explore barriers
Hay & Touyz (2018)	Australia	n/a	Severe and enduring anorexia nervosa (SE-AN)	Persistent state of dietary restriction, underweight and over evaluation of weight/ shape with functional impairment. Duration of ≥3 years of AN Exposure to at least 2 evidence-based treatments delivered appropriately with a diagnostic assessment and formulation that incorporates the persons eating disorder health literacy and stages of change	Aim to better define SEED using their clinical expertise and the literature base.	Identify 3 main areas: 1) Persistent, unrelenting symptoms, 2) of long duration (at least 3 years), 3) treatment resistance (accurate formulation and received all evidence-based treatments)
Hsu & Lieberman (1982)	USA	8 pts chronic anorexia	Chronic anorexia	≥ 5 years with no remission of ≥ 4 months, low weight, amenorrhea of 6 months or loss of libido, weight	Used paradoxical intention with pts and followed up 2, 4 years later. Told to try to keep	50% in normal weight at follow up, none remained under psychiatric care

				phobia or pursuit of thinness cognitions, behaviour directed towards losing weight	anorexia and explored reasons for keeping AN	
Le Grange et al. (2014)	USA, UK and Australia	63 pts SE-AN	Severe and enduring anorexia nervosa (SE-AN)	≥ 7 years<br>≤ BMI 18.5	Part of larger study 30 session CBT or SSCM. Looked at moderators and mediators	Reports predictors of QoL at end of treatment including being younger, shorter duration illness and being employed
Noordenbos et al. (1998)	The Netherlands	600 letters written to ED service analysed, 98 were chronic	Chronic AN	≥ 10 years	Qualitative analysis of letters from patients with and without chronic ED.	Chronic had tried more treatments and were negative about them Recommend patients and clinicians being optimistic and focusing on QoL
Raykos et al. (2018)	Australia	134 pts receiving CBT-E as outpatients	Severe and enduring anorexia nervosa (SE-AN)	≥ 7 years	Looked if duration, BMI or severity predict outcomes in CBT-E?	Duration did not predict changes in symptoms or QoL or BMI, pre-treatment EDE severity did not predict outcomes, pre-treatment BMI did not predict outcomes. Re-ran splitting int 7 years+/- but no differences between groups. SE-AN still improve the same as short duration ED
Robinson (2014)	UK	-	SEED (SEED-AN or SEED-BN)	Severity:<br>Require regular monitoring for serious medical or psychological problems	Describes how to define and identify SEED patients and what the clinical features are from biological, psychological and social perspective.	Describes medical implications of long-term ED

				Require regular contact with at least 2 members of ED team<br>Require regular (at least monthly) discussion at MDT<br>Require MDT management review at least every 6 months<br>How enduring:<br>≥7 years	Discusses stigma<br>Discusses treatment of SEED.	
Robinson et al. (2015)	UK	8 patients mix of men and women and in and outpatients	SEED (SEED-AN or SEED-BN)	20 years+ (only wanted "extreme SEED" as unclear about other cut offs)	Interviewed patients	Generated 15 themes about their experience of living with SEED and their treatment
Stiles-Shields et al. (2013)	USA	63 pts SE-AN	Severe and enduring anorexia nervosa (SE-AN)	≥ 7 years	Compare role of therapeutic alliance in CBT-AN vs SSCM in SE-AN. Secondary analysis of another RCT where pts received 30 sessions of CBT or SSCM.	No difference in ratings of therapeutic alliance between treatments. Early therapeutic alliance predicted treatment outcome for restraint and shape concern but no other ED symptoms, depression symptoms or weight. Suggest can make good therapeutic relationship SE-AN
Stockford et al. (2018)	UK	6 women diagnosis SE-AN	Severe and enduring anorexia nervosa (SE-AN)	≥ 10 years, onset in adolescence or early 20s, now ≥ 30 years of age.	Interviews IPA	7 super ordinate themes – understanding development, experience as functional, negative effects of AN, lack of

						early intervention, cycle of accessing services, negative experience staff attitudes, experience of being with other patients
Tierney & Fox (2009)	UK	53 experts in ED	Chronic AN	Entrenched patterns of food restriction<br>Entrenched "anorexic cognitions"<br>Identity intertwined with anorexia<br>BMI under 17.5	Delphi study – how do professionals define chronic AN?	Created criteria for C-AN mentioned. Less consistent for number of years as arbitrary. Also considered lack of motivation to change. No agreement on number of treatment attempts<br>Also discussed treatment for C-AN but less consistency with this
Touyz et al. (2013)	Australia + UK	63 pts with SE-AN	Severe and enduring anorexia nervosa (SE-AN)	≥ 7 years	RCT CBT-AN or SSCM for pts with SE-AN. 30 sessions over 8 months, focus on treatment QoL not necessarily weight gain. Pre, post 6month and 12 months follow up	Both groups showed sig improvements post treatment and at follow up, CBT-E showed larger reductions in ED symptoms
Treasure et al. (2015)	UK	n/a	SEED	≥ 7 years	n/a	Describes staging model and features of eating disorders in SEED stage and how to try to treat these
Treasure et al. (2015b)	UK	n/a	SEED	≥ 7 years	Systematic literature search	Proposes staging model of eating disorders – high risk, early syndrome, full syndrome, SEED

Westmoreland (2016)	USA	n/a	SEED	6-12 years, BMI below 13, regular attention from an MDT. Physical effects e.g., osteoporosis	n/a	- Discuss what SEED is and describe a ‘harm reduction’ model. Also describes palliative care and futility of treatment in SEED.
Wildes et al., (2017)	USA	355 pts with AN diagnosis	SE-AN	Severity better than length of illness when finding subgroups of AN	Aim to create empirical definition of SEED, used structural equation modelling,	Found no evidence for a sub-group of chronic ED, instead split into low and high severity , were chronic in each group
Yager (2020)	USA	n/a	SE-AN	≥ 8 years despite receiving multiple evidence-based treatments by different clinicians	Discuss cases he has worked with	Consider when to stop treating AN. Describe patients who aim for ‘autonomy’ rather than weight loss, even if involving palliative care or maintaining very low BMI
Zhu et al. (2020)	Australia	n/a	SE-AN	Persistent dietary restriction, underweight, over evaluation of weight / shape, functional impairment, > 3 years AN, exposure to 2+ previous evidence-based treatments	Review of treatment trials which include pts with SE-AN	Highlight challenges defining SE-AN and very few specific studies, only 1 RCT
